# Identification of distinct clinical phenotypes and their neurobiological signatures in stress-exposed individuals: A multimodal machine learning approach

**DOI:** 10.1192/j.eurpsy.2026.12225

**Published:** 2026-05-26

**Authors:** Haejin Hong, Hyeonseok Jeong, Yoonji Joo, Youngeun Shim, Yejin Kim, Yunjung Jin, Yejin Choi, Sujung Yoon, In Kyoon Lyoo

**Affiliations:** 1 Ewha Brain Institute, Ewha Womans University, Seoul, Republic of Korea; 2 Department of Brain and Cognitive Sciences, Ewha Womans University, Seoul, Republic of Korea; 3 Graduate School of Pharmaceutical Sciences, Ewha Womans University, Seoul, Republic of Korea

**Keywords:** machine learning, neuroimaging, phenotype, stress-exposed individuals, transdiagnostic clustering

## Abstract

**Background:**

Individual responses to stress are highly heterogeneous, resulting in diverse psychopathological outcomes. This variability poses challenges for traditional diagnostic frameworks and underscores the need for a transdiagnostic approach to guide interventions. This study aimed to identify distinct phenotypes within a stress-exposed population and to characterize their biological profiles using a multimodal machine learning framework.

**Methods:**

A total of 809 stress-exposed adults (mean age 40.5 ± 8.74 years; 53.7% female) underwent clinical, laboratory, and structural MRI assessments. Data-driven clustering of clinical variables identified phenotypes, followed by machine learning classifiers trained on neuroimaging and laboratory data to predict phenotype membership. SHapley Additive exPlanations (SHAP) analysis was used to identify key biological features distinguishing each phenotype.

**Results:**

Three phenotypes were identified: a multi-risk group (*n* = 321) characterized by prominent depression, anxiety, and sleep disturbances; an alcohol-related risk group (*n* = 226) with high alcohol misuse and minimal comorbidity; and a resilient low-risk group (*n* = 262). Machine learning models accurately classified these phenotypes, indicating distinct biological profiles. SHAP analysis revealed phenotype-specific signatures: the multi-risk phenotype was associated with frontal-subcortical structural alterations and dysregulated cortisol, whereas the alcohol-related risk phenotype was characterized by frontal-insular structural alterations and metabolic abnormalities.

**Conclusions:**

This study demonstrates the stratification of stress-exposed individuals into clinically and biologically distinct phenotypes. By integrating multimodal data with machine learning, we identified phenotype-specific neurobiological and metabolic profiles that extend beyond conventional diagnostic frameworks. These findings support a transdiagnostic, data-driven approach to improve risk stratification and inform personalized interventions in stress-exposed populations.

## Introduction

Stress exposure is a well-established precipitant and aggravating factor for a wide spectrum of psychiatric conditions, including depression, anxiety, sleep disorders, and substance use disorders, through its effects on neuroanatomical structures and its modulation of neuroendocrine and immune function [1–3]. Furthermore, individual responses to stress are highly heterogeneous. Some individuals predominantly develop affective symptoms such as depression or anxiety, whereas others manifest behavioral outcomes such as problematic alcohol use or sleep disturbances, even in the context of comparable stressors [1, 3, 4].

Beyond interindividual variability, stress-related psychopathology frequently presents as overlapping and co-occurring conditions rather than discrete disorders. For example, depressive symptoms often co-occur with sleep disturbances, while alcohol misuse is commonly accompanied by anxiety or affective dysregulation [5–8]. This clinical complexity presents a significant challenge for traditional diagnostic frameworks, which rely on categorical boundaries that may obscure underlying, shared pathophysiological processes [9–10]. Consequently, predictions of illness course and the development of timely, targeted interventions remain limited within these conventional nosological systems.

To overcome these limitations, a transdiagnostic perspective has emerged as a critical paradigm in contemporary mental health research [9, 11]. By moving beyond disorder-specific classifications, transdiagnostic approaches seek to identify cross-cutting mechanisms and clinical patterns that contribute to psychopathological vulnerability, irrespective of categorical diagnoses [11–14]. This framework holds promise for advancing risk stratification, enhancing prognostic accuracy, and informing the development of early, personalized interventions in stress-exposed populations.

Aligned with this perspective, the present study aimed to delineate clinically meaningful phenotypes among stress-exposed individuals and to characterize their associated biological signatures using a multimodal machine learning-based approach. Specifically, we employed a two-stage analytical strategy. First, we applied a data-driven, unsupervised clustering algorithm to stress-related symptom profiles – including depression, anxiety, alcohol use, and sleep disturbances – to derive distinct clinical phenotypes. We then developed supervised machine learning models integrating structural magnetic resonance imaging (MRI) and laboratory data to validate these phenotypes and to elucidate their neurobiological signatures.

## Methods

### Participants

This study included 809 adults who reported exposure to multiple categories of stress. Participants ranged in age from 18.6 to 63.9 years (mean = 40.5, SD = 8.74), and 53.7% were female. Participants were recruited from the community through advertisements and public announcements, representing a community-based sample rather than a clinical population. Eligible individuals were those who had experienced at least one stressful life event (SLE) within the past year.

Stress exposure was assessed using a structured self-report checklist designed to capture major categories of recent life stressors. Participants were asked to indicate all SLEs they had experienced during the previous 12 months. The checklist included four predefined categories of stressors with illustrative examples to facilitate accurate reporting: (a) personal and family-related stressors, such as loss of a loved one, divorce or separation, marital conflict, parenting challenges, serious illness or injury of self or family members, caregiving for a sick or elderly family member, infertility, miscarriage, or major family disputes; (b) work and financial stressors, including job loss, unemployment, workplace harassment or conflict, significant financial hardship, bankruptcy, retirement, major work-related transitions, or career setbacks; (c) social and environmental stressors, such as residential relocation, discrimination, social harassment, social isolation, community violence exposure, or major changes in social support networks; and (d) traumatic events, including natural disasters, serious accidents, violence or abuse, witnessing violence, being a victim of crime, physical or sexual assault, life-threatening experiences, or exposure to combat- or war-related events.

The inclusion criteria were intentionally broad, enrolling participants irrespective of current psychiatric disorder status to capture a comprehensive spectrum of stress-related experiences. Exclusion criteria were applied to ensure participant safety and data quality: (a) presence of serious medical or neurological disorders; (b) clinically significant radiological abnormalities detected on MRI; and (c) contraindications to MRI scanning. All study procedures were approved by the Institutional Review Board of Ewha W. University. Written informed consent was obtained from all participants prior to enrollment.

### Clinical and laboratory assessments

Participants underwent comprehensive clinical evaluations. The presence of current psychiatric disorders was assessed using the Structured Clinical Interview for DSM-5 (SCID) [15]. To quantify stress-related psychiatric symptoms, the following standardized instruments were administered: the Hamilton Depression Rating Scale (HDRS) [16] for depressive symptoms, the Hamilton Anxiety Rating Scale (HARS) [17] for anxiety, the Alcohol Use Disorders Identification Test (AUDIT) [18] for alcohol misuse, and the Pittsburgh Sleep Quality Index (PSQI) [19] for sleep disturbances. In addition, psychological traits known to influence stress responses were evaluated using the Connor–Davidson Resilience Scale (CD-RISC) [20] to measure resilience, the Barratt Impulsiveness Scale (BIS) [21] to assess impulsivity, and the Brief-COPE scale [22] to evaluate coping strategies.

Laboratory assessments included serum cortisol and high-sensitivity C-reactive protein (hsCRP) levels, as well as standard blood chemistry panels, complete blood count (CBC), and urinalysis. Blood samples were collected in the morning and processed at a certified clinical laboratory (Green Cross Laboratories, Yongin, South Korea). Serum cortisol levels were measured using a radioimmunoassay (RIA), and hsCRP levels were quantified using an immunoturbidimetric assay. Detailed assay procedures and instrumentation are described in the Supplementary Methods.

### Image acquisition and preprocessing

High-resolution three-dimensional T1-weighted structural images were acquired using a 3.0-Tesla Philips MRI scanner with a 32-channel head coil. Detailed acquisition parameters are provided in the Supplementary Methods. All images were reviewed by experienced radiologists to identify clinically significant abnormalities as part of participant screening.

Image preprocessing was conducted using FreeSurfer software (version 7.2.0) [23]. The standard cortical reconstruction pipeline was implemented [24–26], with cortical parcellation performed using the Destrieux atlas [27] (74 regions per hemisphere) and subcortical segmentation including the hippocampus, amygdala, thalamus, caudate, putamen, pallidum, nucleus accumbens, and brainstem. Full preprocessing procedures are described in the Supplementary Methods.

Bilateral volumes were calculated for each region, averaged across hemispheres, and normalized to intracranial volume [28]. A total of 82 normalized regional brain volumes were derived for subsequent analyses.

### Clustering analysis to identify clinical phenotypes

**Figure 1.**
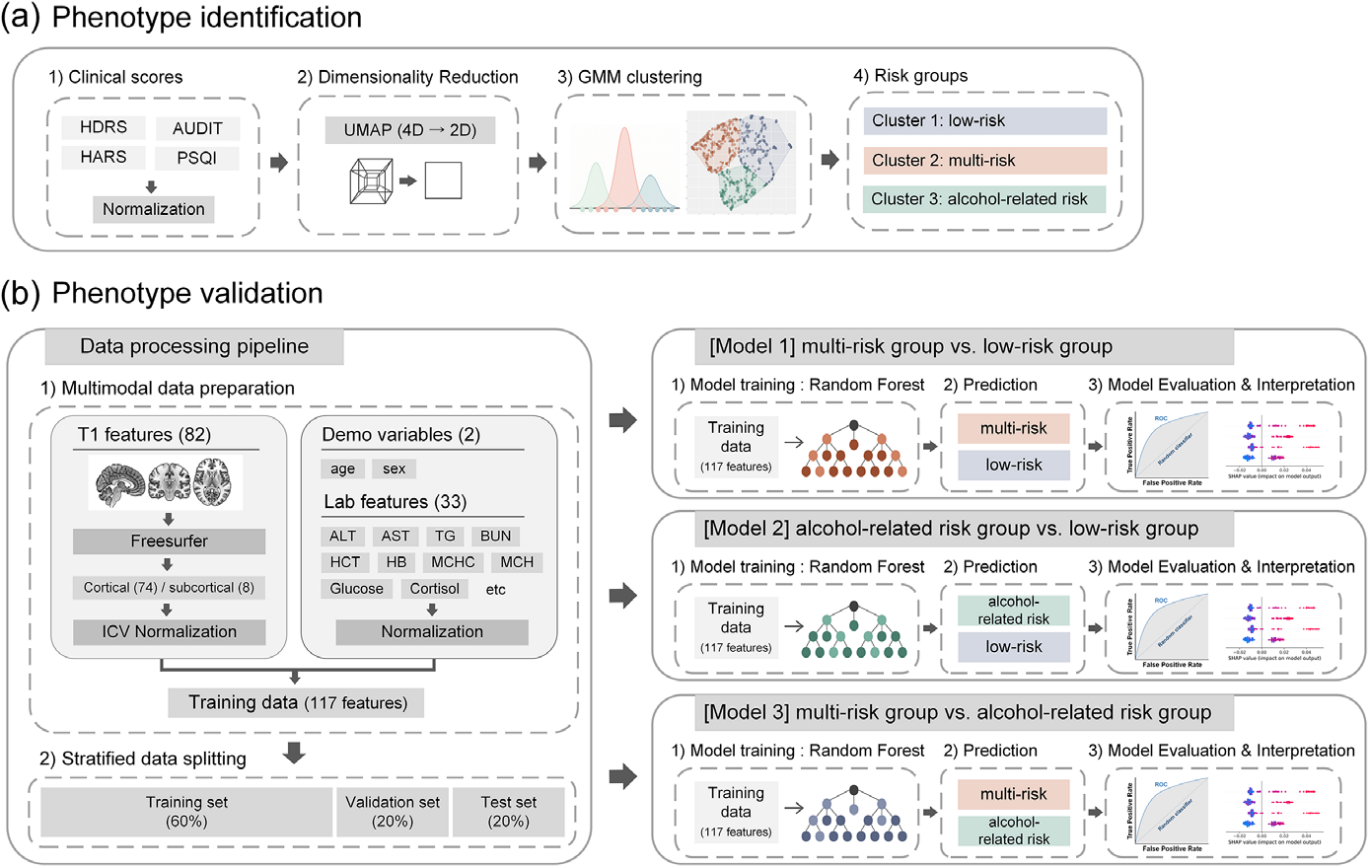
Schematic representation of phenotype identification and validation. (A) Phenotypes in stress-exposed individuals were delineated through unsupervised clustering of stress-related symptom scores (HDRS, HARS, AUDIT, PSQI), employing UMAP for dimensionality reduction and GMM for clustering. (B) Validation of phenotypes was conducted using a machine learning pipeline that integrated multimodal features, including neuroimaging, laboratory, and demographic data. Random forest models were trained, with performance assessed on test datasets and key predictors elucidated through SHAP values. Abbreviations: ALT, alanine aminotransferase; AST, aspartate aminotransferase; AUDIT, Alcohol Use Disorder Identification Test; BUN, blood urea nitrogen; GMM, Gaussian mixture model; HARS, Hamilton Anxiety Rating Scale; HB, hemoglobin; HDRS, Hamilton Depression Rating Scale; HCT, hematocrit; ICV, intracranial volume; MCH, mean corpuscular hemoglobin; MCHC, mean corpuscular hemoglobin concentration; PSQI, Pittsburgh Sleep Quality Index; ROC, receiver operating characteristic; SHAP, SHapley Additive exPlanations; TG, triglycerides; UMAP, uniform manifold approximation and projection. Figure 1. long description.

Clustering analysis was conducted using four standardized clinical measures of stress-related symptoms: HDRS, HARS, AUDIT, and PSQI. All variables were z-score-standardized prior to analysis. To improve visualization of latent structures and enhance clustering efficacy, uniform manifold approximation and projection (UMAP) was applied for nonlinear dimensionality reduction, projecting the data into a two-dimensional space [29]. Gaussian mixture model (GMM) clustering, a probabilistic method that accounts for intracluster variance, was then applied to the UMAP-reduced data [30]. An overview of the clustering workflow is presented in Figure 1. The optimal cluster solution was determined by evaluating multiple validity indices, including the silhouette score, Calinski–Harabasz index, and Davies–Bouldin index [31–33]. GMM solutions for *k* = 2 through 5 were systematically compared to identify the optimal number of clusters.

To evaluate the robustness of the clustering structure, a subsampling stability analysis was additionally performed [34–36]. In this procedure, 80% of the sample was randomly selected without replacement across 1,000 iterations, and cluster assignments from each subsample were compared with the original clustering using the adjusted Rand index (ARI).

To characterize the identified phenotypes, clinical attributes were compared across groups using one-way analysis of variance (ANOVA). Post-hoc pairwise comparisons were adjusted using the Bonferroni correction. A two-tailed p-value <0.05 was considered statistically significant.

### Machine learning-based neurobiological characterization of clinical phenotypes

To elucidate the neurobiological characteristics associated with each clinical phenotype, machine learning-based classification models were developed using multimodal data integrating neuroimaging and laboratory measures. These models were designed to classify participants into their respective phenotypes based on objective biological markers, thereby providing evidence of the neurobiological distinctiveness of each cluster.

The models incorporated a feature set of 117 variables: 82 neuroimaging measures (74 cortical and 8 subcortical volumes), 33 laboratory parameters, age, and sex. A complete inventory of features is provided in Supplementary Table 1. Standardized preprocessing was implemented: z-score normalization was applied to all continuous variables, while the binary variable (coded as 0 or 1) was retained in its original form to preserve categorical status.

The dataset was partitioned using a stratified three-way split to ensure representative distributions of demographic and clinical characteristics across subsets. Stratification was based on sex, age, and cluster assignment. In total, 80% of participants were allocated to the combined training and validation sets, with the remaining 20% reserved for independent testing. The training and validation sets were further divided in a 3:1 ratio, yielding final distributions of 60% training (*n* = 485), 20% validation (*n* = 162), and 20% test (*n* = 162).

Three binary classification models were developed for pairwise discrimination of phenotypes: (a) multi-risk versus low-risk groups; (b) alcohol-related risk versus low-risk groups; and (c) multi-risk versus alcohol-related risk groups. To prevent data leakage, normalization scalers were fitted exclusively on training data and applied to validation and test sets [37]. This preprocessing protocol was consistently applied across all models to ensure comparability.

Random Forest classifiers [38] were used, with hyperparameters optimized separately for each model based on validation performance. Final model evaluation was conducted on the held-out test set using multiple metrics: accuracy, sensitivity, specificity, F1-score, and area under the receiver operating characteristic curve (ROC-AUC) [39], with 95% confidence intervals calculated through 1,000 bootstrap resampling iterations.

Feature importance was examined using SHapley Additive exPlanations (SHAP) values [40]. SHAP is an interpretable machine learning approach that estimates the contribution of each feature to the model’s prediction, allowing identification of variables that most strongly influence classification outcomes. SHAP values were computed for the test set to quantify feature contributions at the individual level. Global feature importance was assessed using the mean absolute SHAP value across participants, while individual-level contributions were also examined to characterize subject-specific prediction patterns.

To further examine the potential influence of sex on classification performance, a sensitivity analysis was conducted in which sex was excluded from the feature set and all models were retrained using identical data partitions and hyperparameters. Additionally, to assess whether neurobiological differences between phenotypes were moderated by sex, linear regression models including a cluster x sex interaction term, adjusted for age, were fitted for key neurobiological features identified through SHAP analysis (top-ranked features, excluding sex). Within-sex regression analyses, adjusted for age, were also performed to confirm that phenotype-specific differences persisted within each sex independently.

## Results

### Identified phenotypes and their clinical characterization

The mean age of the total sample was 40.5 years (standard deviation [SD] = 8.74), and 53.7% of participants were female (Table 1). Among the evaluated solutions (*k* = 2–5), the three-cluster solution demonstrated the strongest overall performance, achieving the highest silhouette score (0.438) and Calinski–Harabasz index (823.3), as well as the lowest Davies–Bouldin index (0.771) (Supplementary Table 2). This solution also exhibited high clustering stability, with a median ARI of 0.932 (IQR = 0.902–0.954) in subsampling analyses, indicating that the identified phenotypes were robust to sampling variability.

**Table 1.** Demographic, clinical, and stress exposure characteristics of participants by cluster Table 1. long description.

Characteristics	Low-risk group(*n* = 262)	Multi-risk group (*n* = 321)	Alcohol-related risk group(*n* = 226)	Total(*n* = 809)	Test
*Demographic characteristics*					
Age	40.4 (7.7)	39.6 (9.4)	42.0 (8.7)	40.5 (8.74)	*F*_2,806_ = 4.92, *p* = 0.008
Female	195 (74.4)	185 (57.6)	54 (23.9)	434 (53.7)	*χ*^2^ = 128.00, *p* < 0.001
*Clinical characteristics*					
HDRS	1.81 (1.65)	8.19 (5.27)	1.92 (1.94)	4.37 (4.75)	*F*_2,806_ = 299.04, *p* < 0.001
HARS	1.25 (1.34)	6.30 (4.91)	1.28 (1.47)	3.26 (4.10)	*F*_2,806_ = 227.76, *p* < 0.001
AUDIT	2.65 (2.29)	7.63 (7.30)	15.15 (6.08)	8.12 (7.53)	*F*_2,806_ = 288.16, *p* < 0.001
PSQI	5.35 (2.09)	11.36 (3.16)	5.92 (2.29)	7.89 (3.84)	*F*_2,806_ = 470.22, *p* < 0.001
*Stress exposure characteristics*					
Personal and family-related stressors	179 (68.3)	133 (41.4)	49 (21.7)	361 (44.6)	*χ*^2^ = 109.00, *p* < 0.001
Work and financial stressors	78 (29.8)	170 (53.0)	175 (77.4)	423 (52.3)	*χ*^2^ = 110.58, *p* < 0.001
Social and environmental stressors	3 (1.2)	16 (5.0)	1 (0.4)	20 (2.5)	*χ*^2^ = 14.18, *p* = 0.001
Traumatic events	97 (37.0)	214 (66.7)	181 (80.1)	492 (60.8)	*χ*^2^ = 102.08, *p* < 0.001

*Note:* Data are presented as *n* (%) or mean (standard deviation). One-way analysis of variance (ANOVA) was used to compare continuous variables across groups, and chi-square test was used to compare categorical variables.

Abbreviations: AUDIT, Alcohol Use Disorder Identification Test; HARS, Hamilton Anxiety Rating Scale; HDRS, Hamilton Depression Rating Scale; PSQI, Pittsburgh Sleep Quality Index.

**Figure 2.**
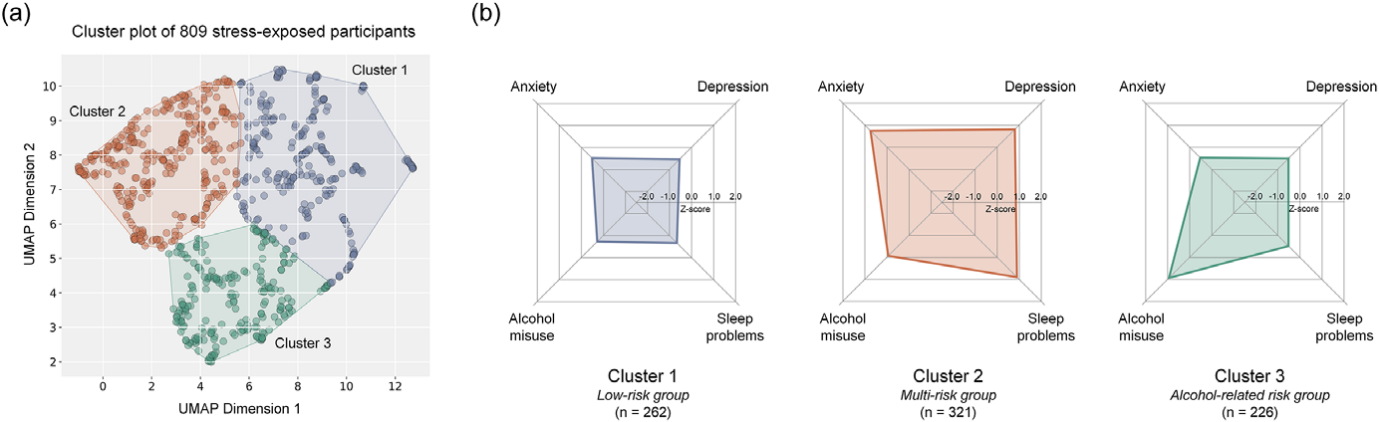
Three stress-related phenotypes with distinct clinical profiles. (A) UMAP visualization demonstrates three stress response clusters, each with characteristic clinical profiles. (B) Cluster 1 represents the low-risk group (*n* = 262), Cluster 2 corresponds to the multi-risk group (*n* = 321), and Cluster 3 represents alcohol-related risk group (*n* = 226). Radar plots illustrate each cluster’s symptom profile across depression, anxiety, alcohol misuse, and sleep disturbance domains. Abbreviations: UMAP, uniform manifold approximation and projection. Figure 2. long description.

The three clusters displayed distinct clinical profiles across depression, anxiety, sleep disturbances, and alcohol misuse (Table 1). Cluster 1 (*n* = 262) was predominantly female and characterized by generally low levels across all four clinical domains; this group was designated the *low-risk group.* Cluster 2 (*n* = 321) consisted of the youngest participants, with a female proportion lower than in Cluster 1 but higher than in Cluster 3. Clinically, this group exhibited elevated levels of depression, anxiety, and sleep disturbances, along with relatively low-to-moderate alcohol misuse; it was designated the *multi-risk group.* Cluster 3 (*n* = 226) included the oldest participants, with a predominance of males, and was characterized by high levels of alcohol misuse, while depression, anxiety, and sleep disturbances remained low; this group was designated the *alcohol-related risk group.* The clinical profiles of these three clusters are summarized in Table 1 and visualized in Figure 2.

Consistent with these defining characteristics, the multi-risk group showed elevated rates of mood disorders (*n* = 53, 16.5%) and anxiety disorders (*n* = 53, 16.5%), whereas the alcohol-related risk group demonstrated a higher prevalence of alcohol use disorders (*n* = 27, 11.9%) (Supplementary Table 3).

Stress exposure characteristics across clusters are presented in Table 1. Traumatic events were the most frequently endorsed category overall (60.8%), followed by work and financial stressors (52.3%), personal and family-related stressors (44.6%), and social and environmental stressors (2.5%). The distribution of stressor types differed significantly across phenotypes (all *p* ≤ 0.001). The alcohol-related risk group reported the highest rates of traumatic events (80.1%) and work and financial stressors (77.4%), while the low-risk group was predominantly characterized by personal and family-related stressors (68.3%). Furthermore, exposure to multiple stressor categories was significantly more prevalent in the two high-risk groups (alcohol-related risk: 78.8%; multi-risk: 62.6%) than in the low-risk group (35.5%; *χ*^2^ = 97.45, *p* < 0.001), indicating a gradient of cumulative stress burden across phenotypes.

Psychological traits also differed across phenotypes (Supplementary Table 4). Overall, the multi-risk group exhibited lower resilience, higher impulsivity, and greater reliance on dysfunctional coping strategies compared with the low-risk group. The alcohol-related risk group also showed elevated dysfunctional coping relative to the low-risk group. Detailed statistical results are provided in the Supplementary Results.

### Model performance to classify identified phenotypes

To assess the discriminative performance of the classification models, three pairwise classification analyses were performed. Model 1 (multi-risk vs. low-risk) demonstrated modest performance, with a ROC-AUC of approximately 0.72 and an accuracy of 65%, indicating some difficulty in distinguishing between these clinically overlapping groups. Model 2 (alcohol-related risk vs. low-risk) showed more robust performance (ROC-AUC = 0.75, accuracy = 74%), with balanced sensitivity and specificity. Model 3 (multi-risk vs. alcohol-related risk) achieved the highest overall performance (ROC-AUC = 0.78, accuracy = 73%), suggesting greater neurobiological differentiation between these two high-risk groups.

Overall, these findings indicate that multimodal features enabled meaningful discrimination between phenotypes, with particularly strong separability between the multi-risk and alcohol-related risk groups, and relatively weaker separability between the multi-risk and low-risk groups. A detailed summary of all performance metrics is available in Supplementary Table 5.

To further examine the robustness of these findings, sensitivity analyses were conducted to evaluate whether the identified neurobiological signatures were substantially explained by sex differences. Excluding sex from the feature set had minimal impact on classification performance across all three models (ΔROC-AUC ≤ 0.011; Supplementary Table 6). Regression analyses including a cluster x sex interaction term, adjusted for age, revealed that the interaction was non-significant for the majority of key neurobiological features (13 of 15 tests), indicating that cluster effects were largely consistent across sexes (Supplementary Table 7). Within-sex analyses further confirmed that the primary neuroanatomical features distinguishing each phenotype were largely preserved in both males and females (Supplementary Table 8).

### Neurobiological characteristics of identified phenotypes

**Figure 3.**
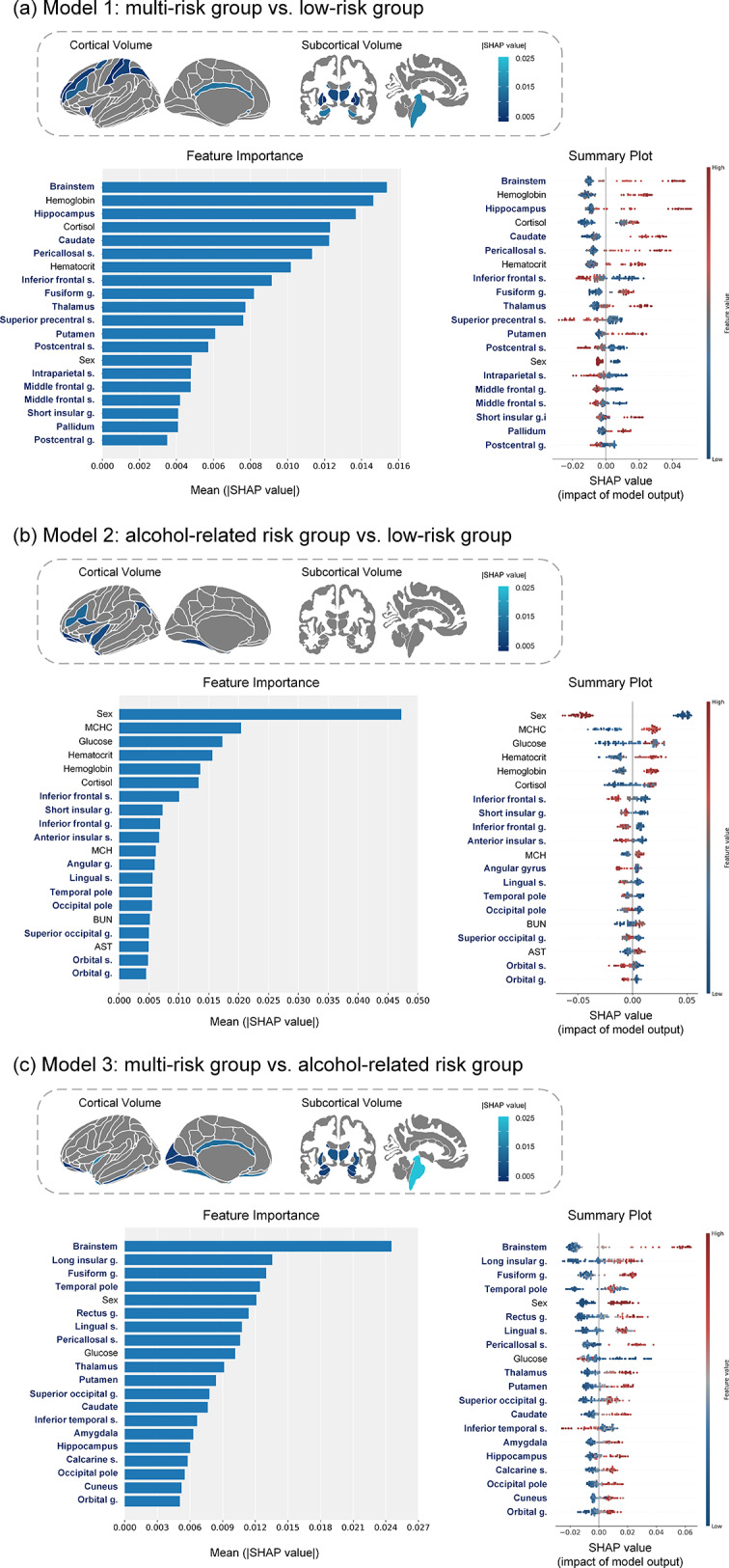
SHAP analysis of feature importance and impact for cluster groups. SHAP feature importance and impact patterns are shown for pairwise classification models of clinical phenotypes: (A) multi-risk group vs low-risk group, (B) alcohol-related risk group vs low-risk group, and (C) multi-risk group vs alcohol-related risk group. Left panels depict mean absolute SHAP values representing feature importance, while right panels display SHAP value distributions colored by feature values. Abbreviations: g, gyrus; s, sulcus; SHAP, SHapley Additive exPlanations. Figure 3. long description.

Comprehensive SHAP analysis revealed clinically meaningful neurobiological distinctions among the identified phenotypes based on integrated neuroimaging and laboratory markers (Figure 3).

In Model 1 (multi-risk group vs. low-risk group), the multi-risk group was primarily distinguished by structural alterations in subcortical and frontal regions, along with dysregulation of stress hormone levels. Specifically, increased volumes in subcortical structures involved in emotion and memory regulation (e.g., brainstem, hippocampus, thalamus) and reduced volumes in frontal regions associated with cognitive control were characteristic of this group. Elevated cortisol levels – a central hormone in the physiological stress response – emerged as the major influential contributor, consistent with a state of chronic physiological stress.

In Model 2 (alcohol-related risk group vs. low-risk group), the alcohol-related risk group was differentiated by sex, clinical chemistry markers linked to alcohol use, and structural brain alterations. Male sex, along with elevated aspartate aminotransferase (AST) and blood urea nitrogen (BUN) levels, were strong predictors. Additional contributing factors included elevated glucose levels and mean corpuscular hemoglobin concentration (MCHC). Neuroanatomically, reduced volumes in the inferior frontal and insular regions – key nodes in circuits governing decision-making and addiction – were central discriminators, reflecting structural brain changes associated with alcohol misuse.

In Model 3 (multi-risk group vs. alcohol-related risk group), the alcohol-related risk group exhibited a distinct neurobiological profile marked by widespread volume reductions in subcortical and temporal regions. Specifically, significant volume loss in the brainstem, thalamus, and hippocampus characterized this group. Additionally, male sex and elevated glucose levels increased the likelihood of classification into the alcohol-related risk group. By contrast, the multi-risk group was relatively associated with reduced volume in the inferior temporal sulcus, suggesting that the two high-risk phenotypes may arise from distinct neurobiological pathways.

## Discussion

The present study employed a transdiagnostic, data-driven methodology to stratify stress-exposed adults into clinically and biologically distinct phenotypes. This approach combined unsupervised clustering of symptom profiles with supervised machine learning classification based on multimodal neuroimaging and laboratory data. Three phenotypes were identified: a resilient low-risk group with minimal symptomatology, a multi-risk group characterized by a constellation of internalizing symptoms, and an alcohol-related risk group dominated by alcohol misuse. The machine learning models achieved moderate to good classification accuracy, and SHAP analyses highlighted phenotype-specific neurobiological signatures. These included subcortical-frontal volumetric alterations and cortisol dysregulation in the multi-risk phenotype, and frontal-insular atrophy coupled with metabolic abnormalities in the alcohol-related risk phenotype. Collectively, these findings extend the current understanding of heterogeneous stress responses and demonstrate the utility of machine learning in delineating transdiagnostic mechanisms that can inform risk stratification and guide tailored interventions.

The “multi-risk” phenotype, defined by the co-occurrence of elevated depression, anxiety, and sleep disturbances, reflects a prototypical internalizing spectrum of psychopathology [41–43]. This clinical constellation aligns with a substantial body of literature documenting the high comorbidity and bidirectional relationships among these disorders, which are increasingly conceptualized as diverse manifestations of a shared underlying vulnerability [43–45]. The psychological traits associated with this phenotype – including low resilience, heightened impulsivity, and greater reliance on dysfunctional coping strategies – further delineate a profile marked by diminished capacity to regulate emotional and cognitive responses to stressors [46–48].

Importantly, this clinical constellation is supported by a distinct neurobiological signature. The identification of elevated cortisol as a principal distinguishing feature provides direct evidence of hypothalamic–pituitary–adrenal (HPA) axis dysregulation, a cornerstone mechanism in the pathophysiology of chronic stress and mood disorders [49–50]. Altered cortisol levels, a recurrent finding in meta-analyses of depression, are indicative of impaired feedback regulation within the HPA axis and reflect a state of sustained physiological allostatic load [51–53].

Structural brain alterations associated with the “multi-risk” phenotype present a particularly informative profile, characterized by reduced volumes in frontal regions alongside increased volumes in key subcortical structures, including the brainstem, hippocampus, and thalamus. The reduction in frontal volumes is consistent with established models of mood and anxiety disorders, which implicate dysfunction within frontal-subcortical circuits that are critical for executive functions such as emotional regulation and cognitive control [54]. Our finding of increased subcortical volumes appears to contradict a large body of literature consistently reporting hippocampal volume reductions in chronic stress and depression, typically attributed to the neurotoxic effects of prolonged glucocorticoid exposure [55, 56]. This apparent discrepancy may stem from the unique characteristics of our sample. Whereas previous research has largely focused on cohorts with chronic, severe depression, our study includes a broader population of stress-exposed individuals who are, on average, younger and present with less severe symptoms. Therefore, the observed volume increase may reflect a state-dependent phenomenon, representing an “intermediate stage” in the pathophysiological response to stress. This raises the possibility of a transient, compensatory hypertrophy in response to heightened allostatic load [57–59]. Ultimately, this result suggests that the brain’s structural response to stress may be nonlinear, highlighting the critical need for longitudinal research to map these dynamic changes over time.

In contrast, the “alcohol-related risk” phenotype represents a distinct, predominantly male, externalizing pathway for coping with stress, characterized by high levels of alcohol misuse with minimal comorbid internalizing symptoms. This separation is consistent with dimensional models of psychopathology that differentiate between internalizing and externalizing spectra [60–61]. The neurobiological profile of this group provides compelling convergent evidence for a well-defined syndrome of alcohol-related brain and systemic pathology.

The defining neuroanatomical alterations – reduced volumes in the inferior frontal and insular regions – are highly consistent with the established neurobiology of alcohol use disorder [62–64]. Volumetric reductions and functional impairments in prefrontal cortical areas, including the inferior frontal gyrus, are among the most consistently reported findings in chronic alcoholism, providing a structural basis for deficits in executive control and decision-making [62, 65]. Similarly, the insula – a critical hub for interoception, craving, and integration of bodily states with emotional experience – is a key node within the neurocircuitry of addiction [66–68]. Volume loss in these frontal-limbic structures offers a robust biological substrate for hallmark clinical features of alcohol use disorder, including impaired self-control and the compulsive drive to consume alcohol [62–65].

Furthermore, the machine learning models identified a coherent metabolic fingerprint for this phenotype, with elevated levels of AST, BUN, and glucose serving as key predictors. These findings are not incidental but represent a systemic signature of the physiological consequences of chronic heavy alcohol consumption [69–71]. Elevated AST reflects hepatocellular stress, increased BUN may indicate alcohol-related dehydration or altered renal function, and dysregulated glucose metabolism is a recognized systemic effect of chronic alcohol use. The ability of the classification model to achieve high accuracy by integrating both central nervous system (structural brain alterations) and peripheral (metabolic markers) data provides strong convergent validity. This demonstrates that data-driven clustering identified a biologically coherent disease state manifesting across multiple physiological systems, underscoring the robustness of this phenotype.

This study exemplifies the practical application of the National Institute of Mental Health’s Research Domain Criteria (RDoC) framework by beginning with dimensional quantification of psychopathology and systematically integrating diverse units of analysis, ranging from neural circuits to peripheral physiological markers [72]. By employing this multilayered, data-driven methodology, the research directly addresses key limitations of categorical diagnostic systems – most notably high comorbidity rates and etiological heterogeneity – by focusing on physiological and clinical continua rather than rigid disorder boundaries [73–74].

Importantly, the phenotypes derived through this approach were discernible even within undiagnosed, general populations. This observation suggests that neurobiological and behavioral biotypes previously identified in patient cohorts are also evident subclinically, potentially preceding the onset of overt clinical syndromes [75–78]. Such findings reinforce the continuous, spectrum-like nature of mental health conditions and underscore their value as early risk markers. The capacity to identify these biotypes in subclinical or community samples highlights the potential for early detection and preventive stratification, a critical advancement for timely intervention and improved outcomes [79].

To further evaluate the robustness of these findings, a subsampling stability analysis demonstrated high clustering stability (median ARI = 0.932), indicating that the phenotypic structure is reliably recovered under repeated resampling and is not dependent on a particular sample composition. While this internal stability does not directly establish cross-population generalizability, several features of the current study provide indirect support for the broader applicability of these phenotypes. Specifically, the use of a community-based sample with broad inclusion criteria enabled the capture of a wide spectrum of stress-related presentations, rather than a narrowly defined clinical subgroup. Furthermore, the convergence of these phenotypes with established dimensional models of psychopathology (i.e., internalizing–externalizing spectra) [60–61], together with consistent validation across clinical, psychological, and neurobiological domains, suggests that the identified clusters reflect meaningful phenotypic heterogeneity rather than sample-specific artifacts. Nevertheless, replication in independent and demographically diverse cohorts will be essential to fully establish the generalizability of these phenotypes across different populations and cultural contexts.

A key methodological strength of this study lies in its two-stage analytical strategy. First, unsupervised clustering enabled hypothesis-free discovery of latent clinical phenotypes. Subsequently, supervised machine learning, combined with SHAP-based model interpretation, advanced the analysis beyond classification to provide explanatory models of the neurobiological features driving phenotypic separation. This represents a crucial step forward, as it transforms otherwise “black box” classifications into specific, neurobiologically grounded hypotheses for each phenotype. In doing so, the approach bridges the gap between data-driven discovery and mechanistic understanding, addressing a common limitation wherein brain-based subtypes demonstrate only weak correspondence with clinically meaningful features.

Notably, while the identified phenotypes exhibited differences in sex distribution – consistent with well-documented sex-related variations in stress response pathways – sensitivity analyses confirmed that the neurobiological signatures were not primarily attributable to sex. Classification models excluding sex maintained comparable performance. Interaction analyses revealed that cluster effects did not significantly vary by sex for the majority of neurobiological features, and within-sex analyses demonstrated that the primary neuroanatomical features distinguishing each phenotype were largely preserved in both males and females. These findings suggest that sex may influence the distribution of phenotypes, but is unlikely to fully account for the core neurobiological distinctions that define them.

Several limitations should be acknowledged. First, the cross-sectional design precludes the determination of causal relationships between neurobiological features and clinical phenotypes. Longitudinal studies are required to establish whether the observed biological distinctions represent predisposing vulnerabilities, consequences of chronic stress exposure, or adaptive responses to ongoing stressors. Second, while the broad inclusion criteria for stress exposure increase generalizability, and we partially addressed this by examining the distribution and cumulative burden of different stressor types across clusters, more detailed characterization of stress exposure – temporal dynamics (e.g., precise timing and duration), severity, and chronicity – was not available in the current dataset. Future studies incorporating more comprehensive measures of stress exposure – including its temporal dynamics, severity, and chronicity – are needed to further clarify their contributions to phenotypic heterogeneity. Third, although integration of multimodal data is a strength, inclusion of genetic, epigenetic, and environmental measures would further enrich the understanding of risk. Finally, while the sample size was substantial, replication in independent cohorts is essential to confirm generalizability across diverse populations and cultural contexts, as the composition and characteristics of phenotypes may vary with demographic factors and stress exposure patterns.

In conclusion, this study demonstrates the value of a transdiagnostic, data-driven approach in addressing the profound heterogeneity of stress-related psychopathology. By integrating clinical, neuroimaging, and laboratory data, we identified and biologically validated three distinct phenotypes, each characterized by unique neurobiological and metabolic signatures. The multi-risk phenotype was defined by a dysregulation of central stress response systems, including the HPA axis and frontal-subcortical circuits, whereas the alcohol-related risk phenotype exhibited a discrete profile of frontal-limbic alterations and systemic metabolic dysfunction consistent with chronic alcohol neurotoxicity. These findings move beyond traditional diagnostic boundaries, providing a more biologically grounded understanding of individual differences in vulnerability to stress. This framework holds significant promise for advancing risk stratification, refining prognostic models, and guiding the development of personalized interventions for individuals facing the adverse consequences of stress.

## Supporting information

10.1192/j.eurpsy.2026.12225.sm001 Hong et al. supplementary material Hong et al. supplementary material

## Data Availability

The data are not publicly available due to ethical restrictions related to participant privacy and consent but are available upon reasonable request and ethics approval.

## Supplementary material

The supplementary material for this article can be found at http://doi.org/10.1192/j.eurpsy.2026.12225.

## [Figure 1] Long description

A two-panel schematic flowchart illustrating the study’s analytical pipeline. Panel (a) depicts the phenotype identification stage: four stress-related symptom scores (Hamilton Depression Rating Scale, Hamilton Anxiety Rating Scale, Alcohol Use Disorder Identification Test, and Pittsburgh Sleep Quality Index) are processed through Uniform Manifold Approximation and Projection for dimensionality reduction, followed by Gaussian Mixture Model clustering to derive distinct clinical phenotypes. Panel (b) depicts the validation stage: multimodal features including 82 brain volume measures, 33 laboratory parameters, age, and sex are integrated into Random Forest classification models. Model performance is evaluated on test datasets, and key predictors are identified through SHAP value analysis.

Navigate back to [Figure 1].

## [Figure 2] Long description

A two-panel figure presenting the three identified stress-related phenotypes. Panel (a) shows a Uniform Manifold Approximation and Projection scatter plot in two-dimensional space, with each point representing one participant colored by cluster assignment. Three clearly separated clusters are visible. Panel (b) presents three radar plots, one per cluster, displaying symptom severity across four domains (depression, anxiety, alcohol misuse, sleep disturbance) on radial axes. Cluster 1 (low-risk group, n=262) shows uniformly low values across all four domains. Cluster 2 (multi-risk group, n=321) shows elevated values for depression, anxiety, and sleep disturbance, with low-to-moderate alcohol misuse. Cluster 3 (alcohol-related risk group, n=226) shows markedly elevated alcohol misuse with low values in the other three domains.

Navigate back to [Figure 2].

## [Figure 3] Long description

A three-panel figure presenting SHAP (SHapley Additive exPlanations) analysis for pairwise classification of clinical phenotypes. Each panel corresponds to one binary comparison: panel (a) multi-risk versus low-risk groups, panel (b) alcohol-related risk versus low-risk groups, and panel (c) multi-risk versus alcohol-related risk groups. Within each panel, the left side shows a horizontal bar chart of mean absolute SHAP values ranking feature importance from most to least influential. The right side shows a SHAP summary plot, with horizontal position indicating the SHAP value (impact on prediction) and color indicating the original feature value (red = high, blue = low). Top-ranked features in panel (a) include cortisol, brainstem, hippocampus, and thalamus volumes; in panel (b), male sex, AST, BUN, and inferior frontal/insular volumes; in panel (c), brainstem, thalamus, and hippocampus volumes.

Navigate back to [Figure 3].

## [Table 1] Long description

A table summarizing characteristics of 809 participants stratified into three clusters: low-risk group (n = 262), multi-risk group (n = 321), and alcohol-related risk group (n = 226), with a Total column and a rightmost statistical test column. Rows include demographic variables (age, sex), four clinical symptom scores (Hamilton Depression Rating Scale, Hamilton Anxiety Rating Scale, Alcohol Use Disorder Identification Test, Pittsburgh Sleep Quality Index), and four stressor type categories (personal/family-related, work/financial, social/environmental, traumatic events). Continuous variables are presented as mean with standard deviation, and categorical variables as count with percentage. Group differences were assessed using analysis of variance for continuous variables and chi-squared tests for categorical variables, with test statistics and p-values presented in the rightmost column.

Navigate back to [Table 1].
