# Supplementary material for: Identification of distinct clinical phenotypes and their neurobiological signatures in stress-exposed individuals: A multimodal machine learning approach
Source: Eur Psychiatry. 2026 May 26;69(1):e62. doi: 10.1192/j.eurpsy.2026.12225 (PMC13276726; doi:10.1192/j.eurpsy.2026.12225)
Supplement: Hong et al. supplementary material [file S0924933826122251sup001.docx]

**SUPPLEMENTARY MATERIAL**

Identification of distinct clinical phenotypes and their neurobiological signatures in stress-exposed individuals: a multimodal machine learning approach

**CONTENTS**

**Supplementary Methods**

Image acquisition and preprocessing

Measurement of serum cortisol and hsCRP

**Supplementary Results**

Psychological characteristics across clinical phenotypes

**Supplementary Figures**

Supplementary Figure 1. ROC curves and confusion matrices for pairwise classification models of clinical phenotypes

**Supplementary Tables**

Supplementary Table 1. Complete list of features used in machine learning classification models

Supplementary Table 2. Clustering validity indices across candidate cluster solutions (k = 2–5)

Supplementary Table 3. Comparison of psychiatric disorder prevalence across cluster groups

Supplementary Table 4. Psychological characteristics and coping mechanisms across cluster groups

Supplementary Table 5. Binary classification performance metrics for risk group discrimination models in the test set

Supplementary Table 6. Sensitivity analyses: classification performance excluding sex as a feature

Supplementary Table 7. Sensitivity analyses: cluster × sex interaction on key neurobiological features

Supplementary Table 8. Sensitivity analyses: within-sex regression of key neurobiological features across clusters

**Supplementary Methods**

**Image acquisition and preprocessing**

High-resolution three-dimensional T1-weighted structural images were acquired using a 3.0 Tesla Philips MRI scanner (Philips Healthcare, Best, The Netherlands) with a 32-channel head coil. Imaging parameters were: repetition time (TR) = 7.4 ms; echo time (TE) = 3.4 ms; flip angle = 8°; field of view (FOV) = 224 × 224 mm²; voxel size = 1 × 1 × 1 mm³; slice thickness = 1 mm; and 180 contiguous sagittal slices.

Image preprocessing was conducted using FreeSurfer software (version 7.2.0, http://surfer.nmr.harvard.edu). The standard FreeSurfer cortical reconstruction pipeline ("recon-all") was implemented, including motion correction, skull stripping, intensity normalization, Talairach transformation, segmentation of white matter and cortical/subcortical structures, tessellation of gray–white matter boundaries, automated topology correction, and surface deformation. All segmentation and parcellation outputs underwent systematic visual inspection to ensure accuracy and data quality. Intracranial volume (ICV) was estimated using FreeSurfer's automated procedures.

**Measurement of serum cortisol and hsCRP**

Measurements of serum cortisol were performed using a radioimmunoassay (RIA), with the Cortisol RIA CT kit (AMP 80-R71200, Asbach Medical Products GmbH, Obrigheim, Germany) and quantified on a gamma counter (COBRA 5010 Quantum; Packard, USA). Serum high-sensitivity C-reactive protein (hsCRP) concentrations were determined by an immunoturbidimetric assay using the Cardiac C-Reactive Protein [Latex] High Sensitive kit (ACN 8217; Diagnostica Stago, Asnieres, France), with analyses conducted on a COBAS 8000 c702 Chemistry Autoanalyzer (Diagnostics International, Rotkreuz, Switzerland). All biomarker assays were carried out at Green Cross Laboratories (Yongin, Gyeonggi-do, South Korea) by laboratory personnel blinded to participant data.

**Supplementary Results**

**Psychological characteristics across clinical phenotypes**

Psychological traits—including resilience, impulsivity, and coping strategies—also differed significantly among phenotypes (Supplementary Table 4). Regarding resilience, the multi-risk group exhibited the lowest scores, significantly lower than both the low-risk group (corrected p < 0.001) and the alcohol-related risk group (corrected p < 0.001). In terms of impulsivity, the multi-risk group demonstrated the highest overall levels. Specifically, this group scored significantly higher on attentional impulsivity compared with both the low-risk and alcohol-related risk groups (all corrected p < 0.001). For motor and non-planning impulsivity, the multi-risk group scored higher than the low-risk group (all corrected p < 0.001), but did not differ significantly from the alcohol-related risk group. With respect to coping strategies, the multi-risk group reported significantly lower levels of adaptive coping, including both problem-focused (corrected p < 0.001) and emotion-focused strategies (corrected p = 0.005), compared with the low-risk group. In contrast, the multi-risk group showed significantly higher reliance on dysfunctional coping strategies than the low-risk group (corrected p < 0.001). Furthermore, the alcohol-related risk group also demonstrated greater use of dysfunctional coping strategies compared with the low-risk group (corrected p < 0.001).

**Supplementary Figure 1.** ROC curves and confusion matrices for pairwise classification models of clinical phenotypes


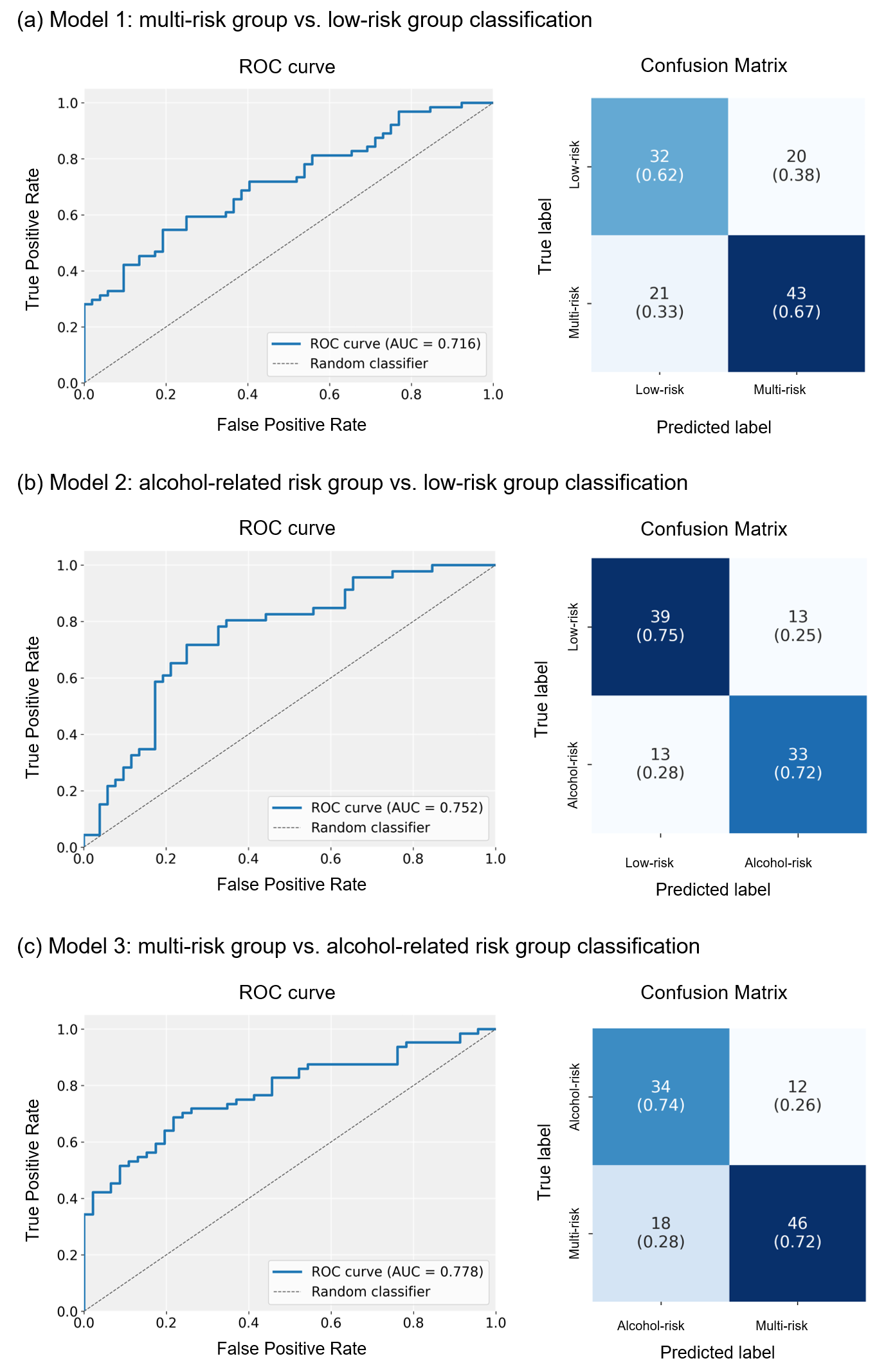


Each panel illustrates classification performance for one of three binary comparisons: (a) multi-risk group vs. low-risk group, (b) alcohol-related risk group vs. low-risk group, and (c) multi-risk group vs. Alcohol-related risk group. ROC curves display AUC values, and confusion matrices present sample counts with proportions in parentheses.

AUC, area under the curve; CI, confidence interval; ROC, receiver operating characteristic.

**Supplementary Table 1.** Complete list of features used in machine learning classification models

| Brain volume measures | |
| --- | --- |
| Cortical features | |
| 1 | Fronto-marginal gyrus and sulcus |
| 2 | Inferior occipital gyrus and sulcus |
| 3 | Paracentral lobule and sulcus |
| 4 | Subcentral gyrus and sulci |
| 5 | Transverse frontopolar gyri and sulci |
| 6 | Cingulate gyrus and sulcus (Anterior part) |
| 7 | Cingulate gyrus and sulcus (Middle-anterior part) |
| 8 | Cingulate gyrus and sulcus (Middle-posterior part) |
| 9 | Cingulate gyrus (Posterior-dorsal part) |
| 10 | Cingulate gyrus (Posterior-ventral part) |
| 11 | Cuneus |
| 12 | Inferior frontal gyrus (Opercular part) |
| 13 | Inferior frontal gyrus (Orbital part) |
| 14 | Inferior frontal gyrus (Triangular part) |
| 15 | Middle frontal gyrus |
| 16 | Superior frontal gyrus |
| 17 | Long insular gyrus |
| 18 | Short insular gyri |
| 19 | Middle occipital gyrus |
| 20 | Superior occipital gyrus |
| 21 | Fusiform gyrus |
| 22 | Lingual gyrus |
| 23 | Parahippocampal gyrus |
| 24 | Orbital gyri |
| 25 | Angular gyrus |
| 26 | Supramarginal gyrus |
| 27 | Superior parietal lobule |
| 28 | Postcentral gyrus |
| 29 | Precentral gyrus |
| 30 | Precuneus |
| 31 | Rectus gyrus |
| 32 | Subcallosal gyrus |
| 33 | Anterior transverse temporal gyrus |
| 34 | Superior temporal gyrus (Lateral aspect) |
| 35 | Superior temporal gyrus (Planum polare) |
| 36 | Superior temporal gyrus (Planum temporale) |
| 37 | Inferior temporal gyrus |
| 38 | Middle temporal gyrus |
| 39 | Lateral sulcus (Horizontal ramus of the anterior segment) |
| 40 | Lateral sulcus (Vertical ramus of the anterior segment) |
| 41 | Posterior ramus of the lateral sulcus |
| 42 | Occipital pole |
| 43 | Temporal pole |
| 44 | Calcarine sulcus |
| 45 | Central sulcus |
| 46 | Cingulate sulcus (Marginal branch) |
| 47 | Anterior insula sulcus |
| 48 | Inferior insula sulcus |
| 49 | Superior insula sulcus |
| 50 | Anterior transverse collateral sulcus |
| 51 | Posterior transverse collateral sulcus |
| 52 | Inferior frontal sulcus |
| 53 | Middle frontal sulcus |
| 54 | Superior frontal sulcus |
| 55 | Sulcus intermedius primus |
| 56 | Intraparietal sulcus |
| 57 | Middle occipital sulcus |
| 58 | Superior occipital sulcus |
| 59 | Anterior occipital sulcus |
| 60 | Lateral occipito-temporal sulcus |
| 61 | Lingual sulcus |
| 62 | Lateral orbital sulcus |
| 63 | Medial orbital sulcus |
| 64 | Orbital sulci |
| 65 | Parieto-occipital sulcus |
| 66 | Pericallosal sulcus |
| 67 | Postcentral sulcus |
| 68 | Precentral sulcus (Inferior part) |
| 69 | Precentral sulcus (Superior part) |
| 70 | Suborbital sulcus |
| 71 | Subparietal sulcus |
| 72 | Inferior temporal sulcus |
| 73 | Superior temporal sulcus |
| 74 | Transverse temporal sulcus |
| Subcortical features | |
| 1 | Thalamus |
| 2 | Caudate |
| 3 | Putamen |
| 4 | Pallidum |
| 5 | Hippocampus |
| 6 | Amygdala |
| 7 | Accumbens |
| 8 | Brainstem |
| Laboratory features | |
| 1 | Cortisol |
| 2 | hsCRP |
| 3 | Albumin |
| 4 | ALT |
| 5 | AST |
| 6 | BUN |
| 7 | Creatine |
| 8 | Glucose |
| 9 | Cholesterol, total |
| 10 | HDL Cholesterol |
| 11 | LDL Cholesterol |
| 12 | Triglyceride |
| 13 | Band Neutrophil |
| 14 | Basophil |
| 15 | Blast |
| 16 | Eosinophil |
| 17 | Lymphocyte |
| 18 | Monocyte |
| 19 | Myelocyte |
| 20 | Neutrophil Segment |
| 21 | HCT |
| 22 | HGB |
| 23 | MCH |
| 24 | MCHC |
| 25 | MCV |
| 26 | PLT |
| 27 | RBC |
| 28 | WBC |
| 29 | Alkaline phosphatase |
| 30 | Bilirubin, total |
| 31 | Protein, total |
| 32 | PH (RU) |
| 33 | Specific Gravity (RU) |
| Demographic variables | |
| 1 | age |
| 2 | sex |

ALT, alanine aminotransferase; AST, aspartate aminotransferase; BUN, blood urea nitrogen; hsCRP, high-sensitivity C-reactive protein; HCT, hematocrit; HGB, hemoglobin; HDL, high-density lipoprotein; LDL, low-density lipoprotein; MCH, mean corpuscular hemoglobin; MCHC, mean corpuscular hemoglobin concentration; MCV, mean corpuscular volume; PLT, platelet; RBC, red blood cell; RU, routine urinalysis; WBC, white blood cell.

**Supplementary Table 2.** Clustering validity indices across candidate cluster solutions (k = 2–5)

| Number of clusters (k) | Silhouette score | Calinski-Harabasz index | Davies-Bouldin index |
| --- | --- | --- | --- |
| 2 | 0.350 | 484.2 | 1.179 |
| 3 | 0.438 | **823.3** | **0.771** |
| 4 | 0.390 | 713.8 | 0.876 |
| 5 | 0.383 | 764.4 | 0.822 |

Clustering validity indices are presented to determine the optimal number of clusters. The Silhouette score and Calinski–Harabasz index indicate better clustering performance with higher values, whereas the Davies–Bouldin index indicates better clustering with lower values. The three-cluster solution demonstrated the highest Silhouette score and Calinski–Harabasz index and the lowest Davies–Bouldin index, supporting the selection of **k = 3** as the optimal cluster solution.

**Supplementary Table 3**. Comparison of psychiatric disorder prevalence across cluster groups

| Psychiatric disorder | Low-risk group  (n = 262) | Multi-risk group  (n = 321) | Alcohol-related risk group  (n = 226) | Total  (n = 809) | Test |
| --- | --- | --- | --- | --- | --- |
| Mood disorder | 4 (1.53) | 53 (16.51) | 3 (1.33) | 60 (7.42) | p < 0.001 |
| Anxiety disorder | 1 (0.38) | 53 (16.51) | 0 (0.0) | 54 (6.67) | p < 0.001 |
| Alcohol use disorder | 0 (0) | 9 (2.80) | 27 (11.95) | 36 (4.45) | p < 0.001 |
| Other psychiatric disorder^a^ | 1 (0.38) | 2 (0.62) | 0 (0.0) | 3 (0.37) | p = 0.784 |

Values represent the number of participants (n) and percentage (%). Participants could be assigned more than one diagnosis. The overall prevalence of having at least one psychiatric diagnosis was 2.3% (n = 6) in the low-risk group, 26.2% (n = 84) in the multi-risk group, and 13.3% (n = 30) in the alcohol-related risk group. Fisher’s exact test was used for statistical comparisons between groups.

^a^ This category includes schizophrenia and eating disorders.

**Supplementary Table 4**. Psychological characteristics and coping mechanisms across cluster groups

| Characteristics | Low-risk group  (n = 262) | Multi-risk group  (n = 321) | Alcohol-related risk group  (n = 226) | Total  (n = 809) | Test |
| --- | --- | --- | --- | --- | --- |
| CD-RISC | 67.89 (15.37) | 56.05 (17.65) | 68.33 (15.68) | 63.32 (17.41) | F_2,806_ = 52.28  p < 0.001 |
| BIS |  |  |  |  |  |
| Attentional impulsivity | 15.89 (2.71) | 17.23 (2.90) | 16.04 (2.73) | 16.46 (2.86) | F_2,806_ = 20.13  p < 0.001 |
| Motor impulsivity | 21.44 (3.84) | 22.21 (3.79) | 22.00 (3.51) | 21.90 (3.74) | F_2,806_ = 3.23  p = 0.040 |
| Non-planning impulsivity | 26.72 (4.03) | 28.12 (4.20) | 27.32 (4.34) | 27.44 (4.23) | F_2,806_ = 8.23  p < 0.001 |
| Brief COPE |  |  |  |  |  |
| Problem-focused strategies | 18.11 (3.26) | 16.95 (3.30) | 17.16 (3.66) | 17.38 (3.43) | F_2,806_ = 9.13  p < 0.001 |
| Emotion-focused strategies | 24.49 (4.50) | 23.35 (4.27) | 23.80 (4.21) | 23.84 (4.35) | F_2,806_ = 5.08  p = 0.006 |
| Dysfunctional coping strategies | 23.05 (4.51) | 26.70 (5.69) | 25.09 (5.15) | 25.07 (5.40) | F_2,806_ = 35.91  p < 0.001 |

Data are presented as mean (standard deviation). One-way analysis of variance (ANOVA) was used to compare continuous variables across groups.

CD-RISC, Connor–Davidson Resilience Scale; Brief COPE, Brief Coping Orientation to Problems Experienced Inventory; BIS, Barratt Impulsiveness Scale.

**Supplementary Table 5.** Binary classification performance metrics for risk group discrimination models in the test set

| Task | ROC-AUC  (95% CI) | Accuracy  (95% CI) | Sensitivity  (95% CI) | Specificity  (95% CI) | F1-Score  (95% CI) |
| --- | --- | --- | --- | --- | --- |
| Model 1: Multi-risk group vs. Low-risk group | 0.72 (0.62-0.80) | 0.65 (0.56-0.73) | 0.67 (0.56-0.79) | 0.62 (0.48-0.75) | 0.68 (0.57-0.76) |
| Model 2: Alcohol-related risk group vs. Low-risk group | 0.75 (0.65-0.84) | 0.73 (0.64-0.82) | 0.72 (0.58-0.84) | 0.75 (0.63-0.87) | 0.72 (0.61-0.81) |
| Model 3: Multi-risk group vs. Alcohol-related risk group | 0.78 (0.68-0.86) | 0.73 (0.64-0.81) | 0.72 (0.60-0.83) | 0.74 (0.60-0.86) | 0.75 (0.66-0.84) |

Binary classification performance is shown for independent test sets. Sensitivity and specificity were calculated with respect to the first-named group as the positive class. All models demonstrated statistically significant discrimination (p < 0.05).

ROC-AUC, area under the receiver operating characteristic curve; CI, confidence interval.

**Supplementary Table 6.** Sensitivity analyses: classification performance excluding sex as a feature

| Task | Full model  ROC-AUC | Sex-excluded model  ROC-AUC | ΔROC-AUC |
| --- | --- | --- | --- |
| Model 1: Multi-risk group vs. Low-risk group | 0.716 | 0.718 | +0.002 |
| Model 2: Alcohol-related risk group vs. Low-risk group | 0.752 | 0.744 | −0.008 |
| Model 3: Multi-risk group vs. Alcohol-related risk group | 0.778 | 0.767 | −0.011 |

The full model includes all features, including sex. The sex-excluded model was trained using the same data splits, preprocessing procedures, and hyperparameters, with sex removed from the feature set. ΔROC-AUC represents the difference between the full and sex-excluded models.

ROC-AUC, area under the receiver operating characteristic curve.

**Supplementary Table 7.** Sensitivity analyses: cluster × sex interaction on key neurobiological features

| Variable |  | Cluster effect | |  | Cluster x sex interaction | |
| --- | --- | --- | --- | --- | --- | --- |
|  |  | β ± SE | p-value |  | β ± SE | p-value |
| Model 1: Multi-risk group vs. Low-risk group | | | | | | |
| Brainstem volume |  | −0.163 ± 0.039 | < 0.001 |  | 0.004 ± 0.065 | 0.956 |
| Hippocampus volume |  | −0.037 ± 0.009 | < 0.001 |  | 0.003 ± 0.010 | 0.768 |
| Thalamus volume |  | −0.061 ± 0.014 | < 0.001 |  | 0.004 ± 0.012 | 0.715 |
| Caudate volume |  | −0.030 ± 0.007 | < 0.001 |  | −0.002 ± 0.005 | 0.697 |
| Cortisol |  | −0.592 ± 0.670 | 0.378 |  | −0.452 ± 0.726 | 0.532 |
| Model 2: Alcohol-related risk group vs. Low-risk group | | | | | | |
| MCHC |  | −0.023 ± 0.115 | 0.840 |  | 0.506 ± 0.202 | 0.013 |
| MCH |  | 0.721 ± 0.194 | < 0.001 |  | 0.226 ± 0.320 | 0.490 |
| Glucose |  | −0.917 ± 3.629 | 0.801 |  | 1.929 ± 3.942 | 0.587 |
| Cortisol |  | 1.238 ± 0.652 | 0.059 |  | −1.332 ± 0.844 | 0.114 |
| Inferior frontal sulcus |  | −0.009 ± 0.004 | 0.060 |  | 0.003 ± 0.006 | 0.658 |
| Model 3: Multi-risk group vs. Alcohol-related risk group | | | | | | |
| Brainstem volume |  | −0.159 ± 0.026 | < 0.001 |  | 0.024 ± 0.063 | 0.700 |
| Thalamus volume |  | −0.059 ± 0.009 | < 0.001 |  | 0.011 ± 0.018 | 0.554 |
| Fusiform volume |  | −0.039 ± 0.006 | < 0.001 |  | 0.007 ± 0.012 | 0.572 |
| Pericallosal volume |  | −0.012 ± 0.002 | < 0.001 |  | 0.001 ± 0.005 | 0.821 |
| Putamen volume |  | −0.045 ± 0.007 | < 0.001 |  | 0.026 ± 0.013 | 0.049 |

Data are presented as regression coefficients (β) ± standard errors (SE). Linear regression models were fitted with cluster assignment as the independent variable and adjusted for age. The cluster effect (β ± SE) represents the regression coefficient for the cluster term in the reference sex group. The cluster × sex interaction term tests whether the association between cluster membership and neurobiological features differs by sex. Variables were selected based on the top-ranked SHAP features (excluding sex) from each classification model. A two-tailed p-value < 0.05 was considered statistically significant.

MCHC, mean corpuscular hemoglobin concentration; MCH, mean corpuscular hemoglobin.

**Supplementary Table 8.** Sensitivity analyses: within-sex regression of key neurobiological features across clusters

| Variable |  | Males | |  | Females | |
| --- | --- | --- | --- | --- | --- | --- |
|  |  | β ± SE | p-value |  | β ± SE | p-value |
| Model 1: Multi-risk group vs. Low-risk group | | | | | | |
| Brainstem volume |  | −0.163 ± 0.039 | < 0.001 |  | −0.151 ± 0.025 | < 0.001 |
| Hippocampus volume |  | −0.037 ± 0.009 | < 0.001 |  | −0.032 ± 0.006 | < 0.001 |
| Thalamus volume |  | −0.061 ± 0.014 | < 0.001 |  | −0.053 ± 0.009 | < 0.001 |
| Caudate volume |  | −0.030 ± 0.007 | < 0.001 |  | −0.031 ± 0.005 | < 0.001 |
| Cortisol |  | −0.592 ± 0.670 | 0.378 |  | −1.044 ± 0.358 | 0.004 |
| Model 2: Alcohol-related risk group vs. Low-risk group | | | | | | |
| MCHC |  | −0.023 ± 0.115 | 0.840 |  | 0.483 ± 0.172 | 0.005 |
| MCH |  | 0.721 ± 0.194 | < 0.001 |  | 0.947 ± 0.292 | 0.001 |
| Glucose |  | −0.917 ± 3.629 | 0.801 |  | 1.013 ± 1.280 | 0.430 |
| Cortisol |  | 1.238 ± 0.652 | 0.059 |  | −0.094 ± 0.516 | 0.855 |
| Inferior frontal sulcus |  | −0.009 ± 0.004 | 0.060 |  | −0.006 ± 0.006 | 0.340 |
| Model 3: Multi-risk group vs. Alcohol-related risk group | | | | | | |
| Brainstem volume |  | −0.159 ± 0.026 | < 0.001 |  | −0.135 ± 0.042 | 0.001 |
| Thalamus volume |  | −0.059 ± 0.009 | < 0.001 |  | −0.048 ± 0.015 | 0.002 |
| Fusiform volume |  | −0.039 ± 0.006 | < 0.001 |  | −0.031 ± 0.010 | 0.003 |
| Pericallosal volume |  | −0.012 ± 0.002 | < 0.001 |  | −0.011 ± 0.004 | 0.007 |
| Putamen volume |  | −0.045 ± 0.007 | < 0.001 |  | −0.019 ± 0.011 | 0.078 |

Data are presented as regression coefficients (β) ± standard errors (SE). Linear regression models were fitted separately within males and females, with cluster assignment as the independent variable and adjusted for age. Variables were selected based on the top-ranked SHAP features (excluding sex) from each classification model. A two-tailed p-value < 0.05 was considered statistically significant.

MCHC, mean corpuscular hemoglobin concentration; MCH, mean corpuscular hemoglobin.
